# The relationship between visual hallucinations, functioning, and suicidality over the course of illness: a 10-year follow-up study in first-episode psychosis

**DOI:** 10.1038/s41537-024-00450-8

**Published:** 2024-03-02

**Authors:** Isabel Kreis, Kristin Fjelnseth Wold, Gina Åsbø, Carmen Simonsen, Camilla Bärthel Flaaten, Magnus Johan Engen, Siv Hege Lyngstad, Line Hustad Widing, Torill Ueland, Ingrid Melle

**Affiliations:** 1 NORMENT, Institute of Clinical Medicine, University of Oslo, Oslo, Norway; 2 NORMENT, Division of Mental Health and Addiction, Oslo University Hospital, Oslo, Norway; 3 Early Intervention in Psychosis Advisory Unit for South East Norway, Division of Mental Health and Addiction, Oslo University Hospital, Oslo, Norway; 4 Department of Psychology, Faculty of Social Sciences, University of Oslo, Oslo, Norway; 5 Division of Mental Health and Addiction, Nydalen District Psychiatric Center, Oslo University Hospital, Oslo, Norway; 6 Department of Child and Adolescent Psychiatry, Division of Mental Health and Substance Use, Diakonhjemmet Hospital, Oslo, Norway; 7 Present Address: NORMENT, Division of Mental Health and Addiction, Oslo University Hospital, Oslo, Norway

**Keywords:** Psychosis, Schizophrenia

## Abstract

Visual hallucinations in psychosis are under-researched despite associations with increased illness severity, functional impairments, and suicidality in the few existing studies. Further, there are no long-term longitudinal studies, making it impossible to conclude if these associations are state or trait phenomena. In the current prospective longitudinal study, 184 individuals with first-episode psychosis were assessed with semi-structured clinical interviews and self-report questionnaires at baseline and 10-year follow-up. Participants were grouped based on lifetime experience of visual hallucinations: before or at baseline (VH+/+), first during follow-up (VH−/+), or never (VH−/−). Associations with functioning, suicide attempts, childhood trauma and other markers of illness severity were tested using multinomial logistic regression analysis. At baseline, the VH+/+ group (37.5%), but not VH−/+ (12.5%), had poorer functioning, higher symptom severity, a lower age at onset, and included more individuals with a history of multiple suicide attempts than the VH−/− group (50%). At follow-up, the VH−/+ group, but not VH+/+, had poorer functioning and higher symptom severity than the VH−/− group. However, the number of participants who committed multiple suicide attempts during the follow-up period was again significantly higher in the VH+/+ group. There was no association with childhood trauma. Hence, visual hallucinations are associated with impaired functioning and higher symptom severity, but only in the short-term. However, visual hallucinations that arise early in the course of illness are a risk indicator for repeated suicide attempts throughout the illness course. These findings highlight the relevance of assessing visual hallucinations and monitoring their development over time.

## Background

In contrast to the vast research on auditory hallucinations in psychotic disorders, comparatively little attention has been devoted to visual hallucinations^1^. This may be because auditory hallucinations appear to be more common in psychotic disorders^2–4^, whereas visual hallucinations have traditionally been linked to organic and neurodegenerative conditions^2,5,6^. However, recent meta-analyses indicate that visual hallucinations are less rare in psychosis than previously assumed, with a weighted mean prevalence of 33% in first-episode psychosis (FEP)^7^ and 27% in schizophrenia^6^. Empirical studies point toward similar or higher prevalence estimates in samples with schizophrenia, schizoaffective disorder, bipolar disorder, and FEP^1–3,5,8^. Accordingly, visual hallucinations are a common and transdiagnostic phenomenon in psychotic disorders.

Visual hallucinations tend to co-occur with hallucinations in other, especially auditory modalities^2,3,5^, and are often experienced as simultaneous-multimodal hallucinations (e.g., talking images)^9^. Notably, they are associated with a range of illness severity markers, including higher global, as well as domain-specific symptom severity^2,10^, a larger number of psychiatric comorbidities^5^, longer time spent in acute psychosis^2^, higher administered doses of antipsychotic medication^2^, and a lower age at onset^5,11,12^. Visual hallucinations have also been linked to decreased cognitive and social functioning^2,11^, and increased suicidality both in individuals with psychosis^2,5^ and in help-seeking adolescents with newly occurred mental health problems^13^.

These associations between visual hallucinations and illness severity markers may be explained by shared genetic and neurodevelopmental vulnerabilities. Specifically, it has been suggested that the same genetic and neurodevelopmental factors that underlie a more severe psychopathology also increase the propensity for visual hallucinations and that visual hallucinations in psychosis may be related to larger detrimental neurodevelopmental changes^4,11,14^. This assumption is supported by findings of remarkably high prevalence rates of visual hallucinations in childhood-onset schizophrenia, a condition that is most often associated with poor long-term outcomes and with more pronounced genetic and neuroanatomical abnormalities^11,15^. Functional and structural brain imaging studies of schizophrenia patients substantiate these interpretations, with aberrations observed in those with visual hallucinations^14,16,17^ and regions implicated in visual hallucinations affected in poor-outcome schizophrenia^4,18,19^. The association between visual hallucinations and a lower age at onset has also been interpreted to support a neurodevelopmental hypothesis of visual hallucinations^5,17^.

Increased suicidality in individuals with psychosis who experience visual hallucinations may then be the consequence of a high subjective illness burden resulting from high levels of symptom severity and functional impairments, both of which have been linked to suicidality in psychosis^20,21^. Additionally, visual hallucinations may induce particularly high levels of psychological distress^5,13^, especially when, as is common, they are simultaneous-multimodal in nature^9^. This, in turn, may contribute to increased suicidal ideation^5,13^. Some authors have suggested that (childhood) trauma may explain the association between visual hallucinations and suicidality^4,5^. In both the general population and psychosis samples, childhood trauma has been linked to suicide attempts^22–24^, as well as to visual hallucinations^1,25,26^. However, findings are inconsistent^25–28^ and the question of whether visual hallucinations are related to childhood trauma and to what extent this could explain their link to suicidality warrants further investigation.

Taken together, these findings suggest that visual hallucinations may be a risk indicator for a more severe illness course, culminating in larger functional impairments and increased suicidal behavior. However, whether their association with functional impairments and suicidality persists over the long-term course of illness is currently unknown. Long-term longitudinal observational studies are needed to examine this question but have to the best of our knowledge not been conducted yet. In a 2-year follow-up study, recent experiences of visual hallucinations assessed at study baseline were related to reduced functioning at 1- but not 2-year follow-up^2^. It is unclear whether these findings generalize to long-term functioning and suicidality, and when assessing lifetime experience of visual hallucinations instead of recent experiences. Given that patients with visual hallucinations might constitute a subgroup characterized by particular genetic and neurodevelopmental vulnerabilities, lifetime experience of visual hallucinations may be more suitable to assess in relation to long-term outcomes.

The current study aims to elucidate the relationship between lifetime experience of visual hallucinations, functioning and suicidality over the long-term course of illness in a sample of FEP patients followed up after ~10 years. The primary aim was to establish whether visual hallucinations are related to increased suicidality and impaired functioning both early (at baseline) and late (at follow-up) in the course of illness. Secondary aims included testing other markers of illness severity and history of childhood trauma for their associations with visual hallucinations and investigating whether such associations could explain the link between visual hallucinations, increased suicidality, and impaired functioning.

## Methods

### Participants

The sample consisted of FEP patients from the Thematically Organized Psychosis (TOP) project, a network project studying schizophrenia spectrum and bipolar disorders with consecutive inclusion of in- and outpatients in the Norwegian regions of Oslo, Østfold, and Innlandet from 2004 to 2012. All patients met the diagnostic criteria for an affective or a non-affective psychotic disorder and had experienced, or were currently experiencing, at least one psychotic episode. None had previously received adequate treatment for psychosis, defined as hospitalization for treatment at a psychosis unit or treatment with antipsychotic medication in an adequate dosage for a period of ≥12 weeks or until remission. Participants were recruited at the start of their first adequate treatment. If they were too psychotic to give informed consent at this point of time, they were allowed to enter the study up to 52 weeks after treatment start^29,30^. The study was approved by the Regional Committee for Medical Research Ethics South East Norway and all participants gave written informed consent before participation.

**Fig. 1:**
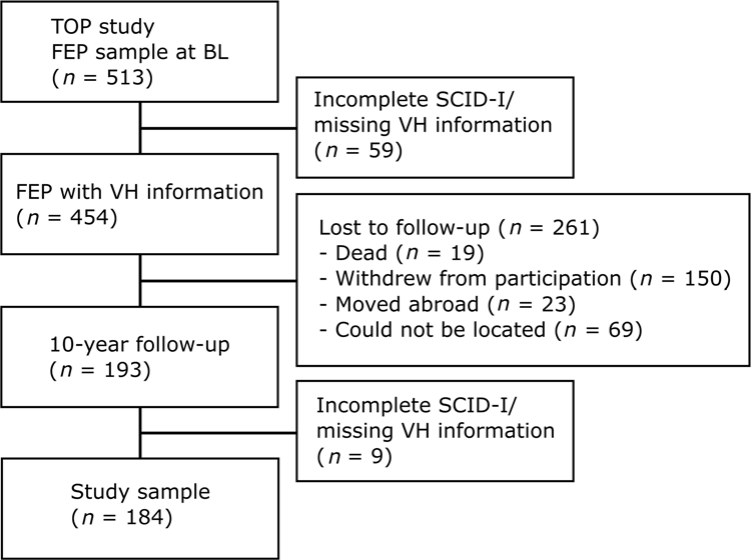
Participant flow and causes of attrition.

Figure 1 presents the selection of the study sample, conditioned on successful follow-up and availability of clinical information. The final sample consisted of 184 participants, assessed at study inclusion (baseline) and ~10 years later (*M* = 9.46, SD = 1.56; follow-up). Follow-up periods differed by site, with *M* = 10.25 (SD = 0.84) years in Oslo (*n* = 138) and *M* = 7.10 (SD = 0.39) years in Innlandet (*n* = 46). A detailed comparison of the study sample with participants lost to follow-up (*n* = 261) is presented in the Supplementary Information, using both baseline characteristics and national health registry data extracted for the follow-up period. This revealed no significant differences in clinical illness course as indicated by contacts with specialized health services during the 10-year follow-up period. However, participants with bipolar disorder as well as participants with a history of multiple suicide attempts at baseline were slightly, and independently of one another, overrepresented in the study sample (Supplementary Information; Table S1). There were no other significant differences in clinical characteristics assessed at baseline, including rates of visual hallucinations.

### Clinical assessments

Trained psychologists or medical doctors conducted all assessments, following a comprehensive semi-structured protocol. Information from participants was further supplemented and validated using medical journals from in- and outpatient treatment facilities.

At baseline, psychiatric diagnoses according to the DSM-IV were confirmed with the Structured Clinical Interview for Mental Disorders (SCID-I)^31^. In addition, basic demographic and clinical information was collected, including age, gender, age at onset (defined as the participant’s age at the start of the first psychotic episode), duration of untreated psychosis (DUP; defined as the number of weeks from the start of the first psychotic episode to the start of the first adequate treatment), and current antipsychotic medication.

Childhood trauma was assessed with the short version of the Childhood Trauma Questionnaire (CTQ)^32^. In analyses, we used the trauma total score as a continuous variable (range 25–125), with higher values indicating more frequent occurrences of traumatic experiences, including emotional, physical, and sexual abuse, as well as physical and emotional neglect. To identify participants with a tendency to minimize and/or deny trauma we used the minimization/denial subscale (MD scale; range 0–3) of the CTQ^33^. Trauma total scores of participants with strong evidence for biased responding (MD score ≥2) were treated as missing in subsequent analyses^34,35^. For 45 participants, the CTQ was not part of the baseline assessment but was administered at follow-up.

At both baseline and follow-up, the SCID-I was used to determine lifetime experience of visual and other hallucinations above the clinical threshold (i.e., a score of three in the SCID). Overall symptom severity was assessed with the total score of the Positive and Negative Syndrome Scale (PANSS; range 30–210)^36^, with higher scores indicating more severe symptoms. Global functioning was assessed with the Global Assessment of Functioning Scale-split version functioning subscale (GAF-F)^37,38^ with scores ranging from 0 (extremely impaired) to 100 (perfect function). GAF-F scores for each participant were discussed and agreed upon in consensus meetings. History of suicide attempts was ascertained both at baseline (history leading up to inclusion in the study) and at follow-up (suicide attempts during follow-up). Since the distribution of this variable was significantly right-skewed, we divided participants into those who had (1) never attempted suicide (“none”), (2) attempted suicide once (“1”), (3) attempted suicide more than once (“>1”) for analyses.

At follow-up, time in active psychosis during the entire 10-year follow-up period was calculated. Using a semi-structured interview with a visual analog timeline, the number of weeks with manifest positive psychotic symptoms (hallucinations, delusions, or unusual thought content, with severity corresponding to PANSS item scores of ≥4) was first determined for each follow-up year and then summarized across years. This variable was treated as missing if information was incomplete. This affected participants from the Innlandet site (due to a shorter follow-up period) and those where time in psychosis could not be recalled and/or derived from clinical journals for every follow-up year.

### Statistical analyses

Participants were divided into three groups (VH-groups): those who reported lifetime experience with visual hallucinations already at baseline (VH+/+), those who did at follow-up only (VH−/+), and those who never experienced visual hallucinations (VH−/−).

To assess the associations between VH-group membership and variables of interest, we applied bivariate multinomial logistic regression models separately for baseline and follow-up variables, with VH-group as the dependent variable. The primary variables of interest were suicidality and functioning. In addition, basic demographic information (gender, age), indicators of illness severity previously linked to visual hallucinations (DUP, PANSS total score, number of psychiatric diagnoses), as well as potential confounders (presence of alcohol or substance abuse or addiction, CTQ trauma total score) were included in the analyses. All analyses involving the PANSS total score were repeated after exclusion of item p3 (“hallucinatory behavior”) to test symptom severity associations independent of recent hallucinations (see Supplementary Information for details). For both baseline and follow-up assessments, all variables with a significant effect in bivariate analyses were subsequently added to a multivariable multinomial logistic regression model to investigate independent associations. Multicollinearity was ruled out by inspection of variance inflation factors. The VH−/− group was the reference group in all multinomial logistic regression analyses. Odds ratios (OR) and 95% confidence intervals (CI) are reported.

The statistical programming language R (R version 4.1.2)^39^ was used for all analyses and all testing was conducted two-sided at a significance level of 0.05.

## Results

Participants had a primary diagnosis within the schizophrenia spectrum (*n* = 84), the bipolar spectrum (*n* = 54), or other psychosis (*n* = 46; Table 1). At 10-year follow-up, 50% of the sample had never experienced visual hallucinations (VH−/−, *n* = 92). The majority of participants with visual hallucinations (37.5%) had had such experiences already at baseline (VH+/+, *n* = 69), and a smaller group (12.5%) at follow-up only (VH−/+, *n* = 23). There were no VH-group differences regarding number of completed follow-up years, *H*(2) = 0.02, *p* = 0.989 (VH+/+: *M* = 9.43, SD = 1.64; VH−/+: *M* = 9.52, SD = 1.50; VH−/−: *M* = 9.47, SD = 1.53).

**Table 1:** Descriptive statistics of baseline variables by VH-group (*N* = 184).

	VH−/− (*n* = 92)		VH+/+ (*n* = 69)		VH−/+ (*n* = 23)	
	*M*	SD	*M*	SD	*M*	SD
Age	28.18	8.91	25.80	8.05	26.52	7.19
Age at onset^a^	24.88	7.86	21.82	7.61	23.17	7.10
DUP^b^	31.00	100.00	52.00	200.25	23.00	137.00
# Diagnoses	1.60	0.91	1.78	1.20	1.74	1.18
PANSS total^c^	58.27	15.64	64.42	17.13	62.74	18.49
CTQ^d^	43.56	13.19	48.83	17.92	40.82	11.27
GAF-F	47.82	12.49	41.93	11.49	44.22	11.58
# Suicide attempts^e^	0.46	0.93	1.52	4.09	0.74	1.25

	*N*	%	*N*	%	*N*	%
Gender (f/m)	39/53	42.4/57.6	39/30	56.5/43.5	12/11	52.2/47.8
Diagnosis						
Bipolar disorder	29	31.5	17	24.6	8	34.8
Other psychosis	29	31.5	17	24.6	0	0.0
Schizophrenia spectrum	34	37.0	35	50.7	15	65.2
Auditory hall. (y/n)^f^	29/63	31.5/68.5	55/13	79.7/18.8	9/13	39.1/56.5
Other hall. (y/n)^g^	16/75	17.4/81.5	34/33	49.3/47.8	4/19	17.4/82.6
Alcohol add./abu. (y/n)	17/75	18.5/81.5	11/58	15.9/84.1	4/19	17.4/82.6
Substance add./abu. (y/n)	16/76	17.4/82.6	13/56	18.8/81.2	5/18	21.7/78.3
Medication: AP						
1. Generation	2	2.2	7	10.1	1	4.3
2. Generation	56	60.9	41	59.4	17	73.9
1. & 2. Generation	3	3.3	4	5.8	0	0.0
None	31	33.7	17	24.6	5	21.7
Medication: Lithium (y/n)	5/87	5.4/94.6	3/66	4.3/95.7	2/21	8.7/91.3
Suicide attempts^e^						
None	66	71.7	44	63.8	14	60.9
One	16	17.4	5	7.2	5	21.7
More than one	9	9.8	18	26.1	4	17.4

*DUP* duration of untreated psychosis, *# Diagnoses* current number of different psychiatric diagnoses, *PANSS total* PANSS total score, *CTQ* minimization-corrected trauma total score of the CTQ, *GAF-F* global functioning score of the GAF scale, *# Suicide attempts* number of lifetime suicide attempts prior to study inclusion, *Bipolar disorder* bipolar I or bipolar not otherwise specified, *Other psychosis* delusional disorder, psychosis not otherwise specified, brief psychotic disorder, or major depression with psychosis, *Schizophrenia spectrum* schizophrenia, schizoaffective disorder, or schizophreniform disorder, *Auditory hall.* lifetime experience of auditory hallucinations, *Other hall.* lifetime experience of hallucinations in other modalities (tactile, olfactory, or gustatory), *Alcohol and Substance add./abu.* presence of an alcohol or substance addiction or abuse disorder, *Medication: AP and Lithium* current antipsychotic or lithium medication.

^a^Missing: *n* = 9 (VH−/−), *n* = 3 (VH+/+).

^b^Missing: *n* = 11 (VH−/−), *n* = 5 (VH+/+), *n* = 1 (VH−/+); values are medians (*M*) and inter-quartile ranges (SD) due to high skewness.

^c^Missing: *n* = 1 (VH−/−), *n* = 2 (VH+/+).

^d^Missing (after exclusion based on MD scale): *n* = 14 (VH−/−), *n* = 16 (VH+/+), *n* = 1 (VH−/+); *n* = 45 cases assessed at follow-up.

^e^Missing: *n* = 1 (VH−/−), *n* = 2 (VH+/+).

^f^Missing: *n* = 1 (VH+/+), *n* = 1 (VH−/+).

^g^Missing: *n* = 1 (VH−/−), *n* = 2 (VH+/+).

### Group differences at baseline

Both the VH+/+ and the VH−/+ group included a larger proportion of participants with a schizophrenia spectrum disorder than the VH−/− group, whereas other psychosis was not represented in the VH−/+ group at all (*χ*^2^ (4) = 12.06, *p* = 0.017; Fisher’s exact test: *p* = 0.005). Rates of bipolar disorder were similar across groups (Table 1). Comorbidities are presented in the Supplementary Information, Table S2. Almost all of the participants with lifetime experience of visual hallucinations at baseline (VH+/+) had also experienced hallucinations in other modalities (80% auditory; 49% tactile, olfactory, or gustatory). Rates of other hallucinations were lower and more similar in the VH−/+ (39% auditory; 17% other) and the VH−/− (32% auditory; 17% other) group (Table 1; association of VH-group with auditory hallucinations: *χ*^2^ (2) = 39.16, *p* < 0.001; Fisher’s exact test: *p* < 0.001; with other hallucinations: *χ*^2^ (2) = 22.22, *p* < 0.001; Fisher’s exact test: *p* < 0.001).

#### Suicidality and functioning

**Fig. 2:**
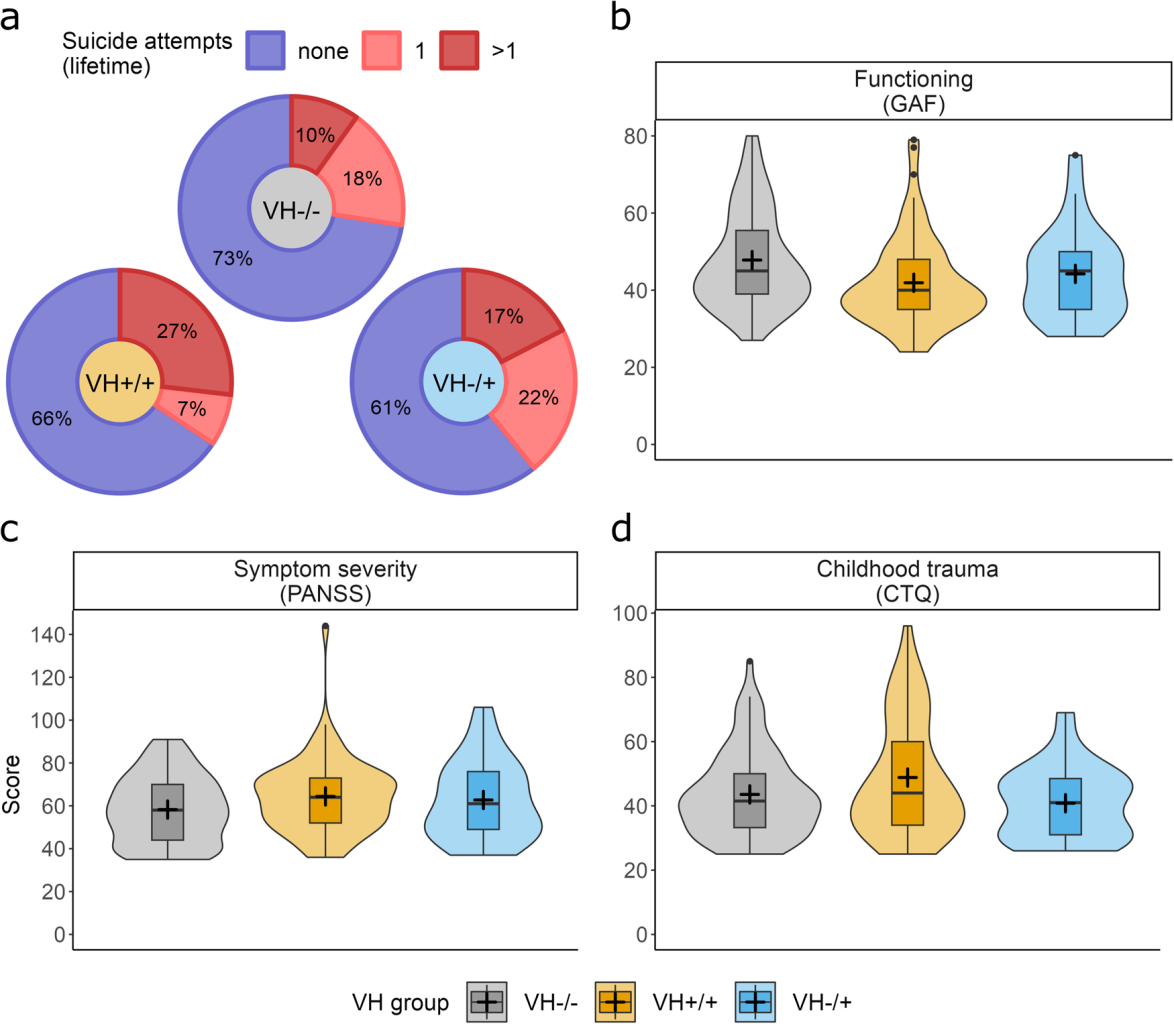
Baseline suicidality, functioning, symptom severity, and childhood trauma scores. **a** Donut plots separate for each VH-group and colored by suicide attempt categories, with percentages calculated based on non-missing values only and rounded to the closest integer. Violin plots showing the distribution of functioning (**b**), symptom severity (**c**), and childhood trauma (**d**) sum scores, colored by VH-group, trimmed to minimum and maximum values in the sample and supplemented with boxplots and mean values (+) for additional information. The whiskers of the boxplots range from the 1. quartile (Q1) and the 3. quartile (Q3) to the lowest/highest observed value respectively, excluding outliers defined by the inter-quartile range (IQR) (bottom: Q1 − 1.5*IQR; top: Q3 *+* 1.5*IQR).

Bivariate multinomial regression analyses revealed significant associations between VH-group status, suicidality, and functioning at baseline. The proportion of participants with a history of multiple, but not single, suicide attempts was significantly larger in the VH+/+ group than the VH−/− group (OR = 3.00, CI [1.24, 7.28], *p* = 0.015; Fig. 2a). Functioning scores were significantly lower in the VH+/+ group, despite a small effect size (OR = 0.96, CI [0.93, 0.99], *p* = 0.003; Fig. 2b). In contrast, neither suicidality nor functioning significantly distinguished the VH−/+ from the VH−/− group (Table 2).

**Table 2:** Bivariate multinomial logistic regression results of baseline variables.

	VH+/+ vs. VH−/−		VH−/+ vs. VH−/−	
Predictor	OR (95% CI)	*p*	OR (95% CI)	*p*
Gender	0.57 (0.30–1.06)	0.077	0.67 (0.27–1.69)	0.399
Age	0.97 (0.93–1.00)	0.080	0.98 (0.92–1.03)	0.419
Age at onset	0.95 (0.90–0.99)	0.019	0.97 (0.92–1.03)	0.373
DUP^a^	1.00 (1.00–1.00)	0.165	1.00 (1.00–1.00)	0.888
# Diagnoses	1.18 (0.88–1.59)	0.273	1.14 (0.74–1.75)	0.544
Alcohol add./abu.	0.84 (0.36–1.92)	0.675	0.93 (0.28–3.08)	0.904
Substance add./abu.	1.10 (0.49–2.48)	0.813	1.32 (0.43–4.08)	0.630
PANSS total	1.02 (1.00–1.04)	0.024	1.02 (0.99–1.05)	0.225
CTQ	1.02 (1.00–1.05)	0.055	0.98 (0.95–1.02)	0.407
GAF-F	0.96 (0.93–0.99)	0.003	0.98 (0.94–1.02)	0.232
Suicide attempts: 1	0.47 (0.16–1.37)	0.167	1.47 (0.46–4.69)	0.512
Suicide attempts: >1	3.00 (1.24–7.28)	0.015	2.10 (0.56–7.78)	0.269

Reference levels of categorical predictors are “female” for gender, “no” for Alcohol and Substance add./abu., and “none” for Suicide attempts (lifetime). Values are rounded to 2 and 3 (*p* values only) decimal places.

*OR* odds ratio, *CI* lower and upper boundaries of the 95% confidence interval, *DUP* duration of untreated psychosis, *# Diagnoses* current number of different psychiatric diagnoses, *Alcohol and Substance add./abu.* presence of an alcohol or substance addiction or abuse disorder, *PANSS total* PANSS total score, *CTQ* minimization-corrected trauma total score of the CTQ, *GAF-F* global functioning score of the GAF scale.

^a^Due to a strongly right-skewed distribution, results were confirmed with nonparametric bootstrapping, using the “boot” function of the “boot” package (boot version 1.3-28.1)^56,57^ with 10,000 replications (bias-corrected CI VH+/+ vs. VH−/− [1.00, 1.00], VH−/+ vs. VH−/− [1.00, 1.00]).

#### Indicators of illness severity and childhood trauma

There were no significant associations between VH-group status and gender, age, DUP, number of psychiatric diagnoses, or rates of alcohol or substance abuse or addiction (Table 2). However, the VH+/+ group had slightly higher symptom severity scores (PANSS total; OR = 1.02, CI [1.00, 1.04], *p* = 0.024; Fig. 2c), as well as a lower age at onset (OR = 0.95, CI [0.90, 0.99], *p* = 0.019) when compared to the VH−/− group. None of these variables significantly distinguished the VH−/+ from the VH−/− group (Table 2). Childhood trauma total scores were slightly, but not significantly, elevated in the VH+/+ group and did not differ between the VH−/+ and the VH−/− group (Fig. 2d and Table 2).

In the multivariable model, only functioning remained significant (OR = 0.95, CI [0.91, 0.99], *p* = 0.020), whereas associations between VH-group and age at onset, symptom severity, and suicidality did not (Supplementary Information, Table S2). Follow-up analyses excluding item p3 (hallucinatory behavior) from the PANSS total score did not indicate substantial differences, although bivariate results differed slightly (Supplementary Information, Table S4).

### Group differences at follow-up

At follow-up, rates of auditory and other hallucinations were higher in all groups compared to baseline, and particularly in the VH−/+ group (Table 3). Rates were similar in the VH−/+ and the VH+/+ group, with almost 90% reporting experience of auditory and almost 70% of other hallucinations in both groups. In the VH−/− group, rates were substantially lower (Table 3; association of VH-group with auditory hallucinations: *χ*^2^ (2) = 35.13, *p* < 0.001; Fisher’s exact test: *p* < 0.001; with other hallucinations: *χ*^2^ (2) = 42.41, *p* < 0.001; Fisher’s exact test: *p* < 0.001).

**Table 3:** Descriptive statistics of follow-up variables by VH-group (*N* = 184).

	VH−/− (*n* = 92)		VH+/+ (*n* = 69)		VH−/+ (*n* = 23)	
	*M*	SD	*M*	SD	*M*	SD
PANSS total^a^	45.70	13.15	46.63	12.90	57.41	24.10
GAF-F^b^	63.79	18.18	59.77	14.50	55.43	18.14
# Suicide attempts^c^	0.20	0.52	0.82	2.71	0.41	1.14
Time in psychosis^d^	140.28	206.40	182.51	202.98	215.53	230.73

	*N*	%	*N*	%	*N*	%
Auditory hall. (y/n)	42/50	45.7/54.3	60/9	87.0/13.0	20/3	87.0/13.0
Other hall. (y/n)	20/72	21.7/78.3	48/21	69.6/30.4	16/7	69.6/30.4
Alcohol add./abu. (y/n)	21/71	22.8/77.2	11/58	15.9/84.1	3/20	13.0/87.0
Substance add./abu. (y/n)	13/79	14.1/85.9	15/54	21.7/78.3	7/16	30.4/69.6
Suicide attempts^c^						
None	77	83.7	51	73.9	18	78.3
One	11	12.0	6	8.7	2	8.7
More than one	3	3.3	10	14.5	2	8.7

*PANSS total* PANSS total score, *GAF-F* global functioning score of the GAF scale, *#* *Suicide attempts* number of suicide attempts during the 10-year follow-up period, *Time in psychosis* number of weeks spent in psychosis during the 10-year follow-up period (missing in case of incomplete data), *Auditory hall.* lifetime experience of auditory hallucinations, *Other hall.* lifetime experience of hallucinations in other modalities (tactile, olfactory, or gustatory), *Alcohol and Substance add./abu.* presence of an alcohol or substance addiction or abuse disorder.

^a^Missing: *n* = 5 (VH−/−), *n* = 4 (VH+/+), *n* = 1 (VH−/+).

^b^Missing: *n* = 2 (VH−/−), *n* = 3 (VH+/+).

^c^Missing: *n* = 1 (VH−/−), *n* = 2 (VH+/+), *n* = 1 (VH−/+).

^d^Missing: *n* = 27 (VH−/−), *n* = 20 (VH+/+), *n* = 6 (VH−/+).

#### Suicidality and functioning

**Fig. 3:**
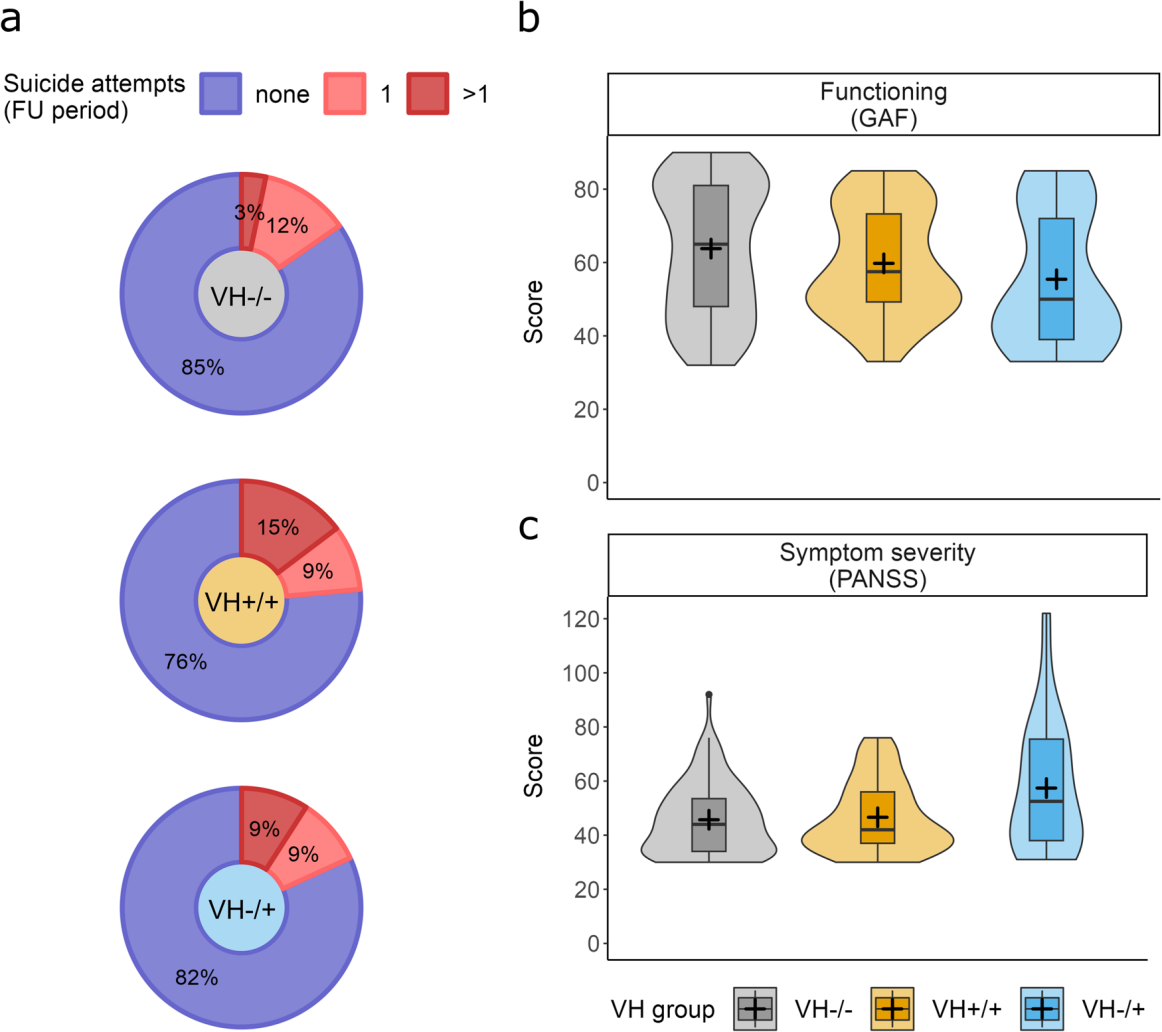
Suicidality, functioning, and symptom severity at follow-up. **a** Donut plots separate for each VH-group and colored by suicide attempt categories, with percentages calculated based on non-missing values only and rounded to the closest integer. Violin plots showing the distribution of functioning (**b**) and symptom severity (**c**) sum scores, colored by VH-group, trimmed to minimum and maximum values in the sample and supplemented with boxplots and mean values (+) for additional information. The whiskers of the boxplots range from the 1. quartile (Q1) and the 3. quartile (Q3) to the lowest/highest observed value respectively, excluding outliers defined by the inter-quartile range (IQR) (bottom: Q1 − 1.5*IQR; top: Q3 *+* 1.5*IQR).

Bivariate multinomial regression analyses revealed significant associations between VH-group status, suicidality and functioning at follow-up. The proportion of individuals who reported multiple suicide attempts during the follow-up period was again higher in the VH+/+ than the VH−/− group (OR = 5.03, CI [1.32, 19.18], *p* = 0.018; Fig. 3a), while the VH−/+ and VH−/− groups did not differ significantly (Table 4). In contrast, functioning scores were slightly lower in the VH−/+ than the VH−/− group (OR = 0.97, CI [0.94, 1.00], *p* = 0.039; Fig. 3b), whereas the VH+/+ and VH−/− groups did not differ significantly (Table 4).

**Table 4:** Bivariate multinomial logistic regression results of follow-up variables.

	VH+/+ vs. VH−/−		VH−/+ vs. VH−/−	
Predictor	OR (95% CI)	*p*	OR (95% CI)	*p*
Alcohol add./abu.	0.64 (0.29–1.44)	0.281	0.51 (0.14–1.87)	0.309
Substance add./abu.	1.69 (0.74–3.83)	0.210	2.66 (0.92–7.71)	0.072
PANSS total	1.00 (0.98–1.03)	0.680	1.04 (1.01–1.07)	0.004
GAF-F	0.99 (0.97–1.00)	0.146	0.97 (0.94–1.00)	0.039
Time in psychosis	1.00 (1.00–1.00)	0.276	1.00 (1.00–1.00)	0.179
Suicide attempts: 1	0.82 (0.29–2.37)	0.719	0.78 (0.16–3.82)	0.757
Suicide attempts: >1	5.03 (1.32–19.18)	0.018	2.85 (0.44–18.34)	0.270

Reference levels of categorical predictors are “no” for Alcohol and Substance add./abu., and “none” for Suicide attempts (follow-up period). Values are rounded to 2 and 3 (*p* values only) decimal places.

*OR* odds ratio, *CI* lower and upper boundaries of the 95% confidence interval, *Alcohol and Substance add./abu.* presence of an alcohol or substance addiction or abuse disorder, *PANSS* PANSS total score, *GAF-F* global functioning score of the GAF scale, *Time in psychosis* number of weeks spent in psychosis during the 10-year follow-up period.

#### Indicators of illness severity

VH-group status was not associated with number of weeks spent in psychosis during the 10-year follow-up period (Table 4), but with symptom severity at follow-up. Here, PANSS total scores were, albeit with a small effect size, significantly higher in the VH−/+ than the VH−/− group (OR = 1.04, CI [1.01, 1.07], *p* = 0.004; Fig. 3c), while the VH+/+ and VH−/− groups did not differ (Table 4).

In multivariable analyses (Supplementary Information), only the association between age at onset and the VH+/+ group remained significant (OR = 0.94, CI [0.89, 0.99], *p* = 0.016), while the link between the VH+/+ group and multiple suicide attempts did not reach statistical significance (OR = 4.34, CI [0.85, 22.23], *p* = 0.078; Table S3). Follow-up analyses excluding item p3 (hallucinatory behavior) from the PANSS total score indicated no substantial differences in bivariate or multivariable results (Supplementary Information, Table S5).

## Discussion

The findings of this study demonstrate that visual hallucinations are a common phenomenon in the course of psychotic disorders, with half of the sample reporting experiences of visual hallucinations during the study period. Furthermore, visual hallucinations were linked to larger functional impairments, higher symptom severity overall, and multiple suicide attempts. Some of these associations were time-point dependent. Specifically, those who had experienced visual hallucinations early in the illness course, that is, at or before baseline (VH+/+ group), showed lower functioning and higher symptom severity at baseline but not at follow-up. In contrast, those who developed visual hallucinations later, that is, during the follow-up period (VH−/+ group), showed lower functioning and higher symptom severity at follow-up but not at baseline. As such, the VH−/+ group seemed to follow a similar, although somewhat delayed course compared to the VH+/+ group. This course was further characterized by the emergence of new hallucinatory experiences in the auditory and other modalities. These findings suggest that experience of visual hallucinations is a risk indicator for ongoing acute illness of greater severity and temporary functional impairments.

Increased symptom severity may result directly from the experience of hallucinatory experiences in the visual and, as indicated by the high co-occurrence rates, additional modalities. These high co-occurrence rates are in line with previous findings^2,3,5,9^ and may partially refer to simultaneous experiences of hallucinations in different modalities (e.g., talking images), which are common among patients with visual hallucinations and have been related to greater conviction of the experiences being real and higher levels of distress^9,40^. Hence, visual hallucinations both add to symptom severity as one of the symptoms considered as well as increase the likelihood for further severe symptoms. While the functional impairments may be caused indirectly by higher levels of symptom severity, robust effects in baseline analyses controlled for symptom severity might also suggest a direct impact of visual hallucinations. In the current study, successful treatment may have diminished the role of hallucinations for symptom severity and functioning over time, given early visual hallucinations (VH+/+) were no longer associated with symptom severity and functional impairments at 10-year follow-up.

Importantly, the presence of severe levels of suicidal behavior, as indicated by rates of multiple suicide attempts, was significantly increased in the VH+/+ group only and remained elevated throughout the follow-up period. Hence, experience of visual hallucinations seems to be a risk indicator for repeated suicide attempts both in the short- and the long-term, but only if visual hallucinations emerged early in the course of illness. The cause for this persistent association between early visual hallucinations and suicidality is unclear but it may be driven by other markers of illness severity related to visual hallucinations. Age at onset, which was significantly lower in the VH+/+ group, has previously been linked to suicidal behavior, though findings are inconsistent^41,42^. Additionally, the elevated symptom severity and functional impairments observed in the VH+/+ group at baseline may reflect a high subjective illness burden, contributing to elevated suicidality^20,21^. Results of the multivariable analyses support this interpretation, with diminished and non-significant associations between visual hallucinations and suicidality once other factors of illness severity were controlled for.

However, the fact that suicidality during follow-up remained increased in this group, despite improvements in functioning and symptom severity, is not in line with this interpretation. Here, several explanations need to be considered. First, and due to how the data was collected, it is unclear precisely when the suicide attempts reported at follow-up took place. They may refer to early phases of the follow-up period, when symptom severity and functional impairment were still elevated in the VH+/+ group. Second, additional factors not measured in this study might contribute to the persistently high rates of suicide attempts in this group. Given that groups only differed regarding the proportion of individuals with a history of multiple and not single suicide attempts, factors previously linked to repeated attempts might be particularly relevant in this context. These include the presence of a borderline personality disorder^43^ or a chronic, non-psychiatric medical illness^44^, and psychological factors such as difficulties in emotion regulation^45^, perceived entrapment^46^, and impulsivity^43^. In borderline personality disorder, hallucinations in the visual and other modalities are not uncommon^47,48^. To what extent the other factors are related to visual hallucinations remains to be investigated. Lastly, anxiety and distress triggered directly by the experience of (visual) hallucinations may increase suicidal ideation and impulsive suicidal behavior^49^.

Childhood trauma was not significantly associated with visual hallucinations and thus less likely to explain their association with suicidality. Previous studies have produced inconsistent results regarding the question of whether visual hallucinations are linked to childhood trauma^1,25,26^ or not^27,28^, presumably due to methodological differences. For example, whereas Solesvik et al.^1^ treated childhood trauma as a binary variable (present or not), Kim et al.^28^ assessed linear relationships between CTQ scores and hallucination severity. The relationship may be non-linear, with any traumatic experience, independent of frequency, increasing the risk for visual hallucinations. This has also been observed in the general population^25^ and may explain the non-significant trend findings in the current study. Alternatively, the relationship may be unique to specific kinds of traumatic experiences (e.g., neglect, but not physical abuse). However, large population-based studies have revealed very little specificity regarding the link between different traumatic experiences and particular psychotic symptoms, with almost all kinds of trauma increasing the risk for almost all kinds of psychotic experiences^25,26,50^.

It has been suggested that shared neurodevelopmental factors underlie both visual hallucinations and a more severe general psychopathology, possibly explaining their interconnection^4,11,14^. The combination of a lower age at onset, higher symptom severity and lower functioning scores observed in the VH+/+ group at baseline provides limited support for this idea. Indeed, individuals in the VH+/+ group might constitute a subgroup of patients characterized by early neurodevelopmental anomalies causing earlier occurrence of first psychotic symptoms, leading to higher symptom severity and detrimental effects on functioning further down the line. However, even if such neurodevelopmental anomalies exist, they do not predestine individuals with visual hallucinations to a more severe illness course, as evidenced by the symptom and functional improvements observed at follow-up. Furthermore, the emergence of new visual (and other) hallucinations in the VH−/+ group does not fit well with a primarily neurodevelopmental perspective. While there is evidence that brain changes such as gray matter loss and lateral ventricular volume increase can manifest both early and late during the course of psychotic disorders^51–53^, the neurodevelopmental anomalies recorded in the context of visual hallucinations refer primarily to changes during prenatal life or early childhood^14,17^. Accordingly, it seems more likely that environmental factors contributed to the late development of visual hallucinations and impacted symptom development and functioning in the VH−/+ group, including stressful life events, insufficient treatment adherence, or substance use during the follow-up period.

The presented findings should be interpreted in the context of some limitations. The reliability and validity of the GAF-F as a measure of functioning have previously been questioned. However, large sample size studies suggest good validity, including in samples with schizophrenia, while reliability is considered acceptable when assessors are trained as they were here^38,54^. Effect sizes for functioning, symptom severity, and age at onset were relatively small. For suicidality, effect sizes were substantially larger, but confidence intervals were wide, introducing some uncertainty around the estimated effects. This was likely caused by small sample sizes in the respective subgroups, and future studies should try to replicate the findings in larger samples. Further, and due to how the data was collected, it is unclear precisely when the reported suicide attempts took place and precisely when visual hallucinations first arose. This limits the extent to which conclusions about the association between newly developed symptoms, suicidality, and current symptom severity (as assessed at baseline and follow-up) can be drawn and does not allow for any causal inferences. In addition, systematically collected information about personality disorders and recent traumatic experiences might have been informative and should be collected in future studies. Finally, participants with bipolar disorder and a history of multiple suicide attempts before baseline were overrepresented in the study sample when compared to those lost to follow-up, perhaps because increased contact with the health care system and a subjective need for help made them easier to recruit for follow-up. Multiple suicide attempt rates observed in this study may thus not represent the general FEP population. However, visual hallucination rates did not differ and there is no reason to assume that the associations between visual hallucinations, functioning, and suicidality would have been different when using the complete sample.

Notwithstanding, the findings of this study shed new light on the interconnection of visual hallucinations, real-life functioning, and suicidality in psychosis. They highlight the importance of routinely assessing the presence of visual hallucinations in clinical settings as a risk indicator for repeated suicide attempts and functional impairments. Here, particular attention should be devoted to those patients who experience visual hallucinations early in the course of illness and have a comparatively low age at onset. Moreover, the development of visual hallucinations over the course of illness should be tracked closely, as the emergence of new hallucinatory symptoms may be a precursor of a less favorable long-term course in terms of functioning and illness severity.

### Supplementary information

Supplementary Information

## Data Availability

Due to ethical restrictions, the data used in the current study are not publicly available. Interested parties may request access from the corresponding author through a reasonable inquiry, subject to approval by the Regional Ethics Committee.

## Code availability

No custom code was used for analyses. Data were analyzed with R (R version 4.1.2)^39^, using the “multinom” function of the “nnet” package (nnet version 7.3-18)^55^ for multinomial logistic regression, the “boot” function of the “boot” package (boot version 1.3-28.1)^56,57^ with 10,000 replications for bootstrapping, and the “ggplot2” package (ggplot2 version 3.4.0)^58^ for plotting.
