# Supplementary material for: The relationship between visual hallucinations, functioning, and suicidality over the course of illness: a 10-year follow-up study in first-episode psychosis
Source: Schizophrenia (Heidelb). 2024 Mar 2;10(1):30. doi: 10.1038/s41537-024-00450-8 (PMC10908818; doi:10.1038/s41537-024-00450-8)
Supplement: Supplementary file 1 — Supplementary Information [file 41537_2024_450_MOESM1_ESM.pdf]

Supplementary Information for:

The relationship between visual hallucinations, functioning, and suicidality over the course of illness: a 10-year follow-up study in first-episode psychosis

Isabel Kreis*[1], Kristin Fjelnseth Wold[1], Gina Åsbø[2], Carmen Simonsen[2,3], Camilla Bärthel Flaaten[2,4], Magnus Johan Engen[5], Siv Hege Lyngstad[5], Line Hustad Widing[2,6], Torill Ueland[2,4], Ingrid Melle[1]

[1]NORMENT, Institute of Clinical Medicine, University of Oslo, Oslo, Norway; [2]NORMENT, Division of Mental Health and Addiction, Oslo University Hospital, Oslo, Norway; [3]Early Intervention in Psychosis Advisory Unit for South East Norway, Division of Mental Health and Addiction, Oslo University Hospital, Oslo, Norway; [4]Department of Psychology, Faculty of Social Sciences, University of Oslo, Oslo, Norway; [5]Division of Mental Health and Addiction, Nydalen District Psychiatric Center, Oslo University Hospital, Oslo, Norway; [6]Department of Child and Adolescent Psychiatry, Division of Mental Health and Substance Use, Diakonhjemmet Hospital, Oslo, Norway

Supplementary Results

Comparison of study participants with those lost to follow-up

Of all participants who were deemed eligible for study participation at baseline and for whom SCID-I based ratings of lifetime experience of visual hallucinations were available (N = 454), 57% (n = 261) were lost to follow-up. Of these, 150 had withdrawn from participation, 69 could not be located, 23 had moved abroad, and 19 had died. For nine out of the remaining 193 followed-up participants, SCID-I information assessed at follow-up was incomplete, and they were therefore excluded from the current study.

To evaluate the representativeness of the final sample included in this study (n = 184), comparisons with those lost to follow-up were conducted. Groups were compared on a range of baseline variables central to the current study. In addition, national health registry data was accessed to compare the amount of time spent in specialized health services during what would constitute a participant's 10-year follow-up period. Dates of contacts with specialized health services were counted as full days and summarized both across different levels of care, and per level of care, with level of care corresponding to either inpatient care, outpatient contacts, or day treatment. Only contacts where the primary reason was due to schizophrenia spectrum diagnoses (ICD-10 codes F20-F29), bipolar disorder (F31) or depressive episodes (F32) were included in this summary. Since the time period covered by the available registry data only ranged from 01.01.2008 to 31.12.2020, information was incomplete for follow-up years falling outside of this time range. Therefore, the average number of days spent in specialized health care per year was calculated based on all follow-up years for which information was complete.

Descriptive and test statistics of baseline variables are presented in Table S1.

Table S1

Descriptive and test statistics of baseline variables by follow-up status

	Lost to follow-up (n = 261)		Study participants (n = 184)		Test statistics[a]	
	M	*SD*	*M*	*SD*	*U*	*p*
Age	28.26	9.19	27.08	8.43	25929.5	.151
Age at onset	24.86	9.31	23.48	7.75	22815.0	.129
DUP[b]	32.00	126.00	36.00	150.00	19672.0	.600
PANSS	61.43	15.77	61.12	16.74	23922.0	.820
GAF-F	44.48	11.50	45.16	12.26	23435.5	.666
	N	%	*N*	%	χ^2	*p*
Gender (f/m)	100/161	38.3/61.7	90/94	48.9/51.1	4.53	.033
Diagnosis						
Bipolar disorder	45	17.2	54	29.3	9.56	.009
Other psychosis	84	32.2	46	25.0		
Schizophrenia spectrum	132	50.6	84	45.7		
Alcohol add./abu. (y/n)	39/222	14.9/85.1	32/152	17.4/82.6	0.32	.573
Substance add./abu. (y/n)	60/201	23.0/77.0	34/150	18.5/81.5	1.06	.303
Suicide attempts					11.67	.003
never	192	73.6	124	67.4		
once	49	18.8	26	14.1		
more than once	18	6.9	31	16.8		
Visual hall. (y/n)	89/172	34.1/65.9	69/115	37.5/62.5	0.41	.524

Note. DUP = duration of untreated psychosis, PANSS total = PANSS total score, GAF-F = global functioning score of the GAF scale, Alcohol and Substance add./abu. = presence of an alcohol or substance addiction or abuse disorder, Visual hall. = lifetime experience of visual hallucinations. Values are rounded to 2 and 3 (p-values only) decimal places.

[a] test statistics are U for continuous variables (Mann-Whitney U tests), and χ^2 (Chi-squared tests) for nominal variables

[b] values are medians (M) and inter-quartile ranges (SD) due to high skewness

The participant group lost to follow-up included significantly more male participants (61.7%) than the final group of study participants (51.1%; Table S1). Rates of bipolar disorder were lower in the group lost to follow-up (17.2%) than in the final study sample (29.3%), and

rates of other psychosis slightly higher (32.2%; Table S1). While there were no differences in lifetime experience of visual hallucinations (Table S1), the percentage of participants with a history of multiple suicide attempts was significantly larger in the final study sample (16.8%) than in the group that was lost to follow-up (6.9%; Table S1).

To assess whether this difference in past suicide attempts could be explained by the differences in gender and bipolar disorder rates, a logistic regression was fitted to predict follow-up status (included in study vs. lost to follow-up) by gender (reference: male), diagnosis (reference: schizophrenia spectrum disorder), and suicide attempts (reference: none). Here, there was no effect of gender, $b = 0.26$, $p = .206$, or of other psychosis, $b = -0.08$, $p = .722$, on follow-up status. However, the effect of bipolar disorder remained significant, $b = 0.70$, $p = .006$, as did the effect of multiple, $b = 1.01$, $p = .002$, though not single suicide attempts, $b = -0.29$, $p = .292$.

This indicates that participants with bipolar disorder and participants with a history of multiple suicide attempts are overrepresented in the final study sample, and that this cannot be explained by higher rates of female participants in this group. Given the absence of group differences in rates of visual hallucinations, this bias does not affect the main analyses of this study, namely the investigation of the association between visual hallucinations, suicidality, and functioning. However, it does mean that the presented rates of multiple suicide attempts may not be representative of the general FEP population.

There were no significant differences in average number of days spent in specialized health care per year within the follow-up period. This was true across levels of care ($U = 25193.5$, $p = .376$; $Md_{\text{study participants}} = 8.58$, $Md_{\text{lost to FU}} = 8.80$), as well as for inpatient care ($U = 26004.5$, $p = .115$; $Md_{\text{study participants}} = 0$, $Md_{\text{lost to FU}} = 1.1$), outpatient care ($U = 24609.5$, $p = .654$; $Md_{\text{study participants}} = 4.75$, $Md_{\text{lost to FU}} = 6.30$), and day treatment ($U = 24041.5$, $p = .966$; $Md_{\text{study participants}} = 0$, $Md_{\text{lost to FU}} = 0$). This indicates a comparable clinical course of illness in the study

sample and the participants lost to follow-up. Number of years excluded from the average calculation due to missing or incomplete registry data did not differ significantly between groups ($U = 25935$, $p = .121$; $Md_{\text{study participants}} = 0$, $Md_{\text{lost to FU}} = 1$).

Psychiatric comorbidities at baseline

Total number of psychiatric comorbidities were similar for all VH groups (see Table 1, main article). An overview of all comorbidities per group is presented in Table S2.

Table S2

Psychiatric comorbidities by VH-group

	VH-/- (n=92)		VH+/+ (n=69)		VH-/+ (n=23)	
diagnosis	***N***	**%**	***N***	**%**	***N***	**%**
Alcohol add./abu.	17	18.48	11	15.94	4	17.39
Substance add./abu.	16	17.39	13	18.84	5	21.74
Bipolar NOS	0	0.00	3	4.35	0	0.00
Borderline personality disorder[a]	1	1.09	0	0.00	0	0.00
Major depressive disorder	10	10.87	13	18.84	2	8.70
OCD	2	2.17	1	1.45	1	4.35
Other psychosis	2	2.17	0	0.00	1	4.35
Phobia	1	1.09	0	0.00	0	0.00
PTSD	1	1.09	1	1.45	0	0.00
Substance induced disorder	1	1.09	0	0.00	0	0.00
Dysthymia	1	1.09	1	1.45	0	0.00

Note. Alcohol and Substance add./abu. = presence of an alcohol or substance addiction or abuse disorder, Bipolar NOS = bipolar not otherwise specified, OCD = Obsessive-compulsive disorder, PTSD = Post-traumatic stress disorder. Single individuals may be represented in multiple diagnosis categories.

[a] Personality disorders were not assessed systematically but only when necessary for differential diagnoses.

Multivariable analyses

To test if other markers of illness severity influenced the associations between VH-group status, functioning, and suicidality, multivariable models were constructed for both baseline (Table S3) and follow-up analyses (Table S4), including all variables with a statistically significant association with VH-group in bivariate analyses.

Table S3

Multivariable multinomial logistic regression including baseline variables

	VH+/+ vs. VH-/-		VH-/+ vs. VH-/-	
predictor	***OR (95% CI)***	***p***	***OR (95% CI)***	***p***
Age at onset	0.97 (0.92-1.02)	.233	0.98 (0.92-1.05)	.505
PANSS total	0.99 (0.96-1.02)	.481	1.00 (0.96-1.04)	.937
GAF-F	0.95 (0.91-0.99)	.020	0.99 (0.93-1.04)	.587
Suicide attempts: 1	0.40 (0.13-1.23)	.111	1.36 (0.41-4.51)	.618
Suicide attempts: >1	1.88 (0.66-5.39)	.239	1.83 (0.43-7.88)	.416

Note. Number of complete cases included (fitted values) = 168

Table S4

Multivariable multinomial logistic regression including follow-up variables

	VH+/+ vs. VH-/-		VH-/+ vs. VH-/-	
predictor	***OR (95% CI)***	***p***	***OR (95% CI)***	***p***
Age at onset	0.94 (0.89-0.99)	.016	0.99 (0.92-1.06)	.782
PANSS total	0.97 (0.94-1.01)	.156	1.03 (0.99-1.08)	.149
GAF-F	0.97 (0.94-1.00)	.078	0.99 (0.95-1.04)	.798
Suicide attempts: 1	0.70 (0.22-2.25)	.551	0.83 (0.15-4.64)	.835
Suicide attempts: >1	4.34 (0.85-22.23)	.078	3.30 (0.42-26.11)	.257

Note. Number of complete cases included (fitted values) = 159

PANSS analyses excluding item p3 (‘hallucinatory behavior’)

The PANSS total score includes ratings of hallucinatory behavior (item p3) without specification by modality. Therefore, and because the PANSS assesses current symptom severity whereas visual hallucinations were assessed in a dichotomized manner and according to lifetime experience, item p3 remained included in the PANSS total score for all main analyses. Nevertheless, here we provide supplementary analyses using a reduced PANSS total score calculated after exclusion of item p3. Results of bivariate and multivariable analyses are presented.

In bivariate analyses at baseline, the reduced PANSS score (Figure S1) was no longer significantly associated with VH-group status, VH+/+ vs. VH-/- group: *OR* = 1.02, CI [1.00 1.04], *p* = .064, VH-/+ vs. VH-/- group: *OR* = 1.02, CI [0.99, 1.05], *p* = .244. Multivariable analysis results did not change, with direction, size, and significance of effects similar to the model including the full PANSS scale (Table S5).

Figure S1

PANSS sum scores at baseline and follow-up, with and without hallucinatory behavior

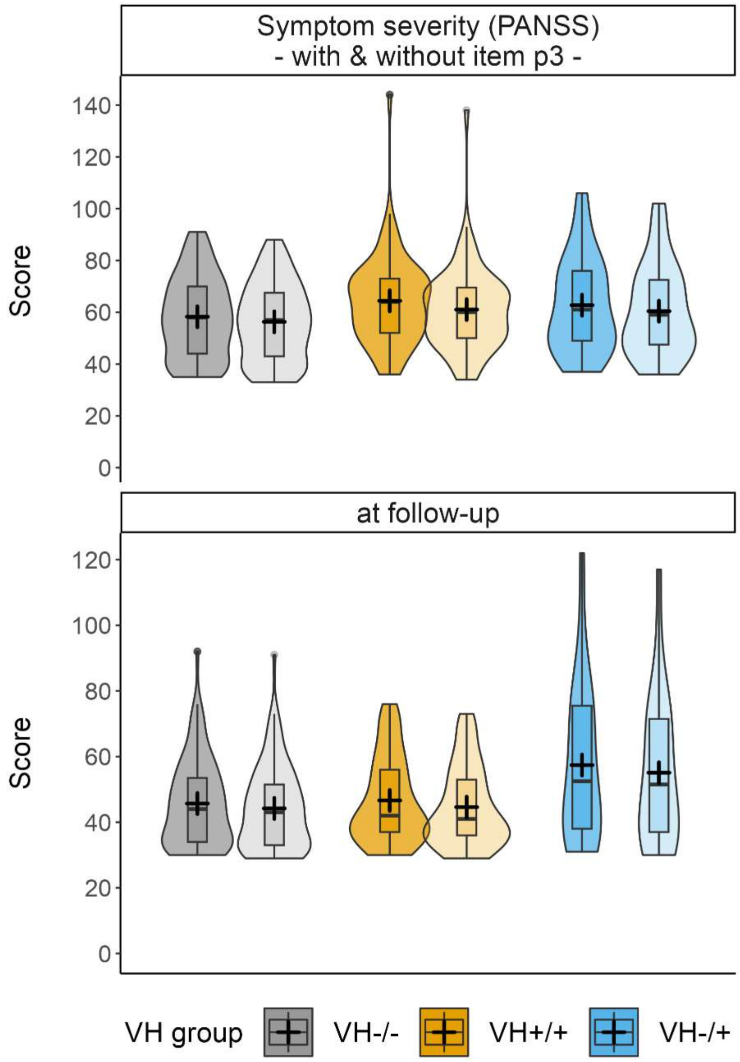

Note. Violin plots showing the distribution of symptom severity scores at baseline (top row) and follow-up (bottom row), trimmed to minimum and maximum values in the sample and supplemented with boxplots and mean values (+) for additional information. Plots are colored by VH-group, with paler shades (right-hand side of each group's plot pair) representing PANSS scores without item p3 ('hallucinatory behavior').

Table S5

Multivariable multinomial logistic regression including baseline variables

	VH+/+ vs. VH-/-		VH-/+ vs. VH-/-	
predictor	***OR (95% CI)***	***p***	***OR (95% CI)***	***p***
Age at Onset	0.97 (0.92-1.02)	.207	0.98 (0.92-1.05)	.517
PANSS (-p3)	0.98 (0.96-1.01)	.265	1.00 (0.96-1.04)	.923
GAF-F	0.95 (0.91-0.99)	.010	0.99 (0.93-1.04)	.595
Suicide attempts: 1	0.39 (0.13-1.19)	.098	1.36 (0.41-4.55)	.614
Suicide attempts: >1	1.81 (0.63-5.21)	.271	1.84 (0.42-7.97)	.415

In bivariate analyses at follow-up, associations between VH-group status and symptom severity remained unchanged when using the reduced as opposed to the total PANSS score, with the VH-/+ group still displaying higher symptom scores than the VH-/- group (*OR* = 1.04, CI [1.01, 1.08], *p* = .005; Figure S1) but no significant difference between the VH+/+ and the VH-/- group (*OR* = 1.00, CI [0.98, 1.03], *p* = .855).

Similarly, there was no change in multivariable analysis results, with direction, size, and significance of effects similar to the model including the PANSS total score (Table S6).

Table S6

Multivariable multinomial logistic regression including follow-up variables

	VH+/+ vs. VH-/-		VH-/+ vs. VH-/-	
predictor	***OR (95% CI)***	***p***	***OR (95% CI)***	***p***
Age at Onset	0.94 (0.89-0.99)	.016	0.99 (0.92-1.06)	.754
PANSS (-p3)	0.97 (0.93-1.01)	.108	1.03 (0.99-1.08)	.175
GAF-F	0.97 (0.94-1.00)	.057	0.99 (0.95-1.04)	.743
Suicide attempts: 1	0.71 (0.22-2.26)	.558	0.82 (0.15-4.56)	.823
Suicide attempts: >1	4.36 (0.85-22.41)	.078	3.24 (0.41-25.61)	.266
